# Supplementary material for: The DUX4–HIF1α Axis in Murine and Human Muscle Cells: A Link More Complex Than Expected
Source: Int J Mol Sci. 2024 Mar 15;25(6):3327. doi: 10.3390/ijms25063327 (PMC10969790; doi:10.3390/ijms25063327)
Supplement: Supplementary file 1 [file ijms-25-03327-s001.zip › ijms-2825412-supplementary.pdf]

Supplementary Data

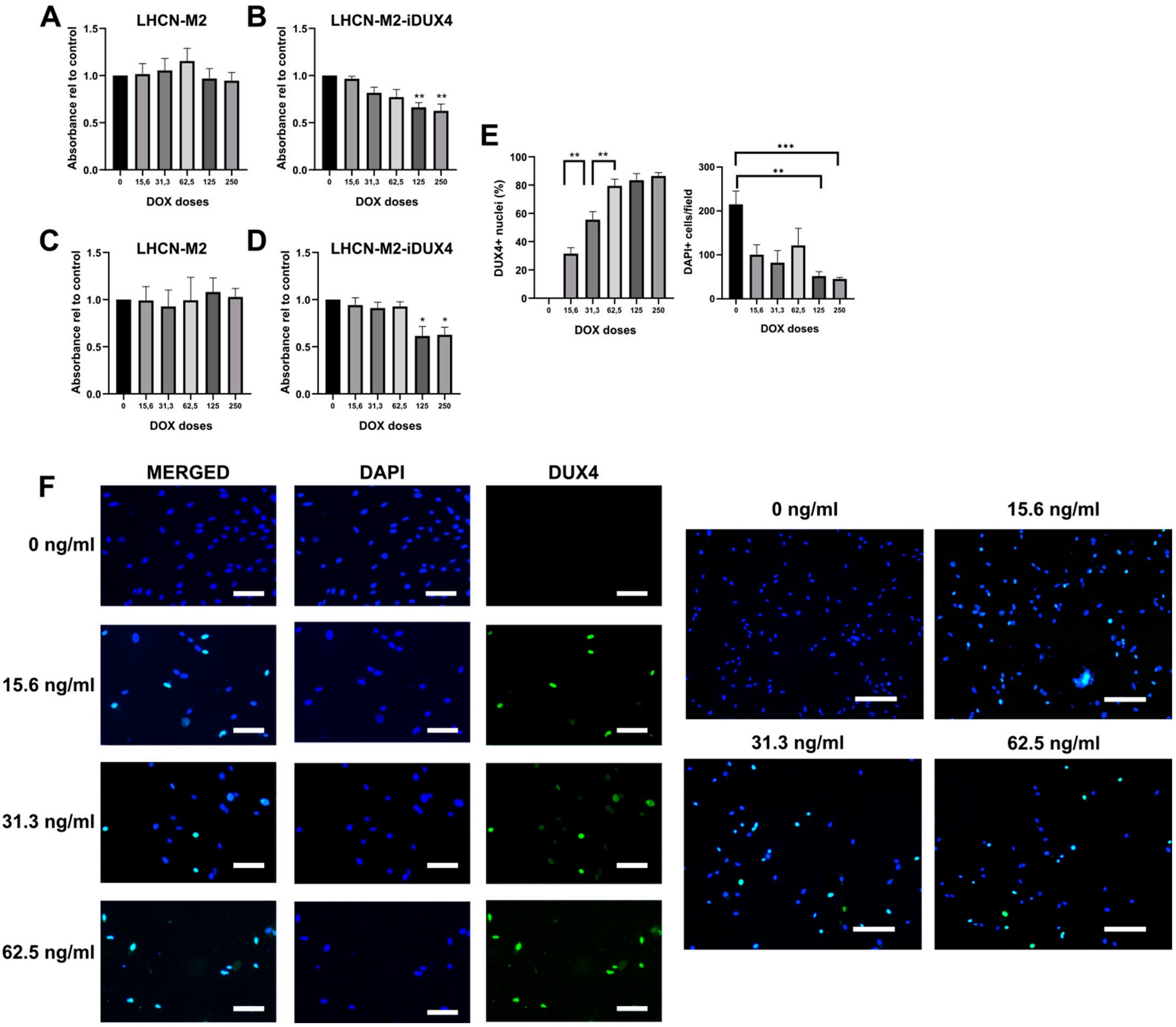

Figure S1. Effect of DUX4 induction on LHCN-M2 and LHCN-M2-iDUX4 cell viability. (**A-D)** MTT (A-B) and CCK8 (C-D) tests were performed 24h after induction of *DUX4* expression with increasing doses of doxycycline (DOX, ng/ml). Mean ± SEM, * $p < 0.05$, ** $p < 0.01$, One-way ANOVA with Holm Sidak post hoc test vs. control (DOX: 0 ng/ml). **(E) Left panel**. Quantification of DUX4 positive (DUX4^{+}) nuclei normalized to the total number of nuclei (DAPI) 24h after induction of *DUX4* expression with increasing doses of DOX. Mean ± SEM, ** $p < 0.01$, One-way ANOVA with Holm Sidak post hoc test. **Right panel**. Quantification of DAPI positive (DAPI+) nuclei per field 24h after induction of *DUX4* expression with increasing doses of DOX. Mean ± SEM, ** $p < 0.01$, *** $p < 0{,}001$, One-way ANOVA with Holm Sidak post hoc test. **(F)** Representative field showing DUX4^{+} nuclei detected by immunofluorescence (green) as described in Figure 1. DAPI was used to stain nuclei (blue). Left panel scale bar = 100μm, Right panel scale bar = 50 μm. Experiments were performed on 3 independent cultures, each in triplicate. The total number of counted cells is on average, 4719 for myoblasts.

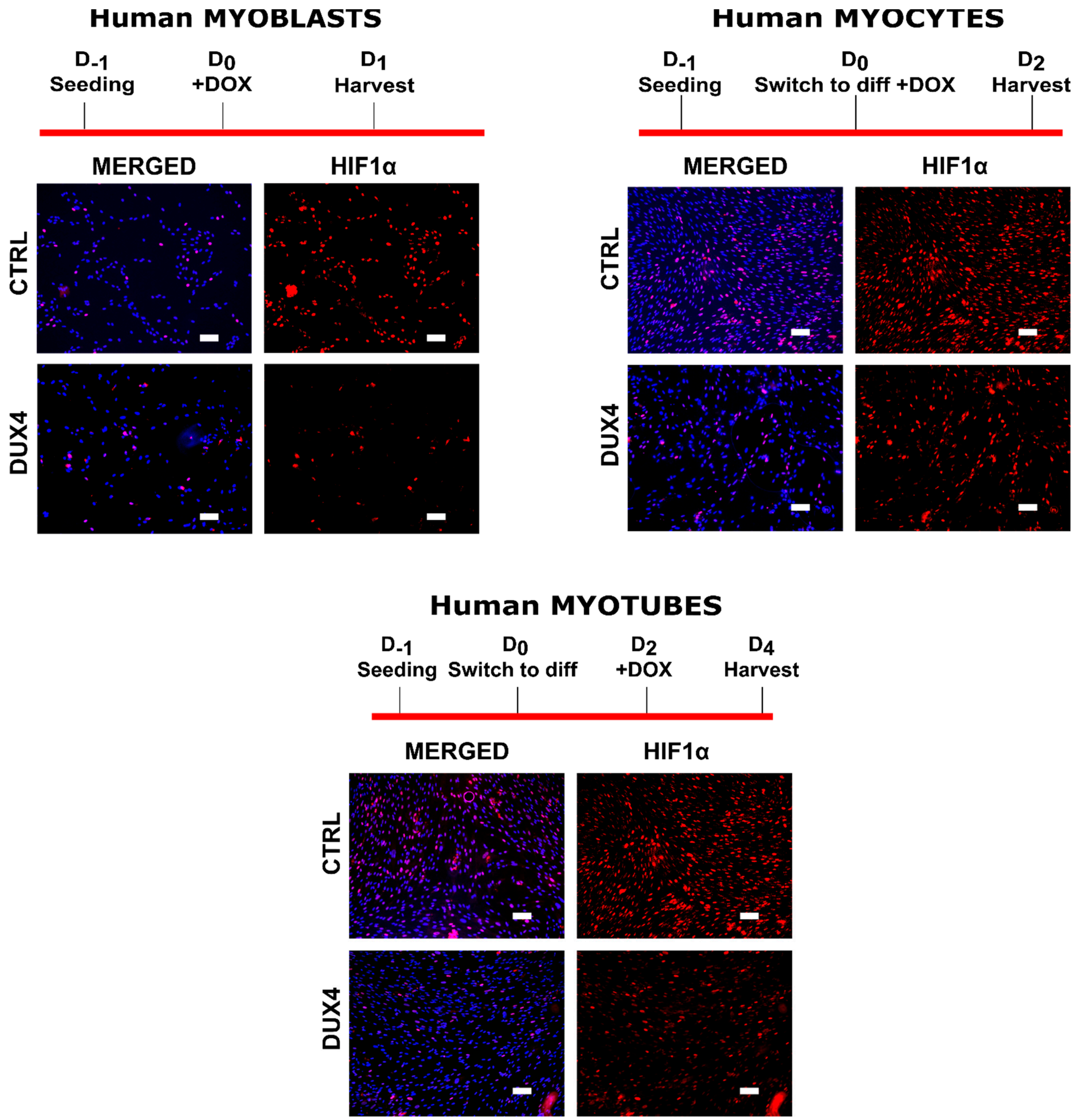

Figure S2. Effect of DUX4 on the percentage of HIF1α^{+} nuclei in human LHCN-M2-iDUX4 muscle cells. Representative pictures of large fields used for HIF1α positive (HIF1α^{+}) nuclei (red IF) quantifications presented in Figures 1 and S3. DAPI was used to visualize nuclei (blue). Scale bar = 100µm.

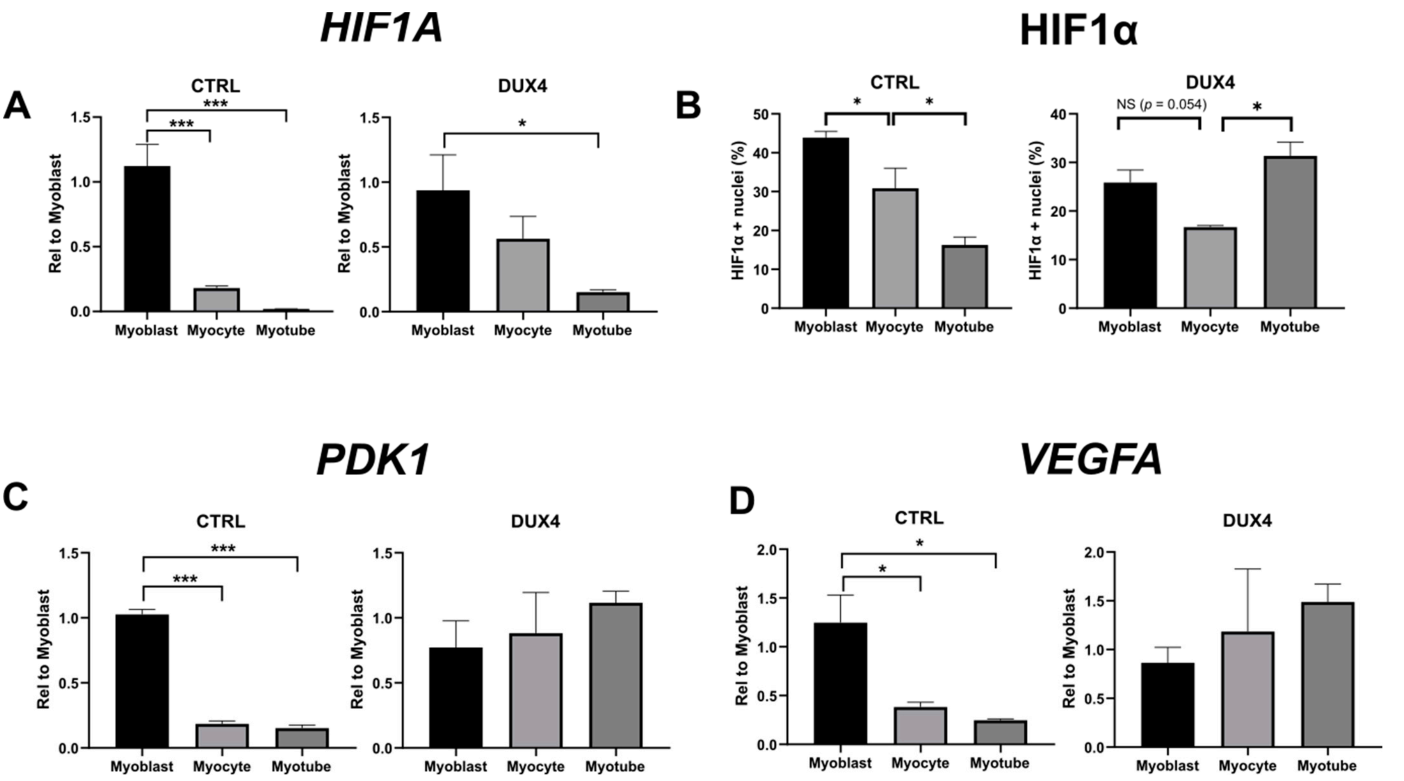

Figure S3. Comparison of HIF1α expression pattern in human LHCN-M2-iDUX4 myoblasts, myocytes and myotubes. Cell culture, induction of DUX4 expression by doxycycline and myogenic differentiation were performed at a standard PO_2 of 21% as in Figure 1. **(A, C, D)** Relative *HIF1A*, *PDK1* and *VEGFA* mRNA level quantified by RT-qPCR normalized to *RPLP0.* Mean ± SEM, * $p < 0.05$, *** $p < 0.001$, One way ANOVA with Holm Sidak post hoc test. N=3. **(B)** Quantification of HIF1α^+ nuclei normalized to the total number of nuclei (DAPI staining). Mean ± SEM, * $p < 0.05$, One way ANOVA with Holm Sidak post hoc test, N=3. All experiments were performed on 3 independent cultures, each at least in triplicate.

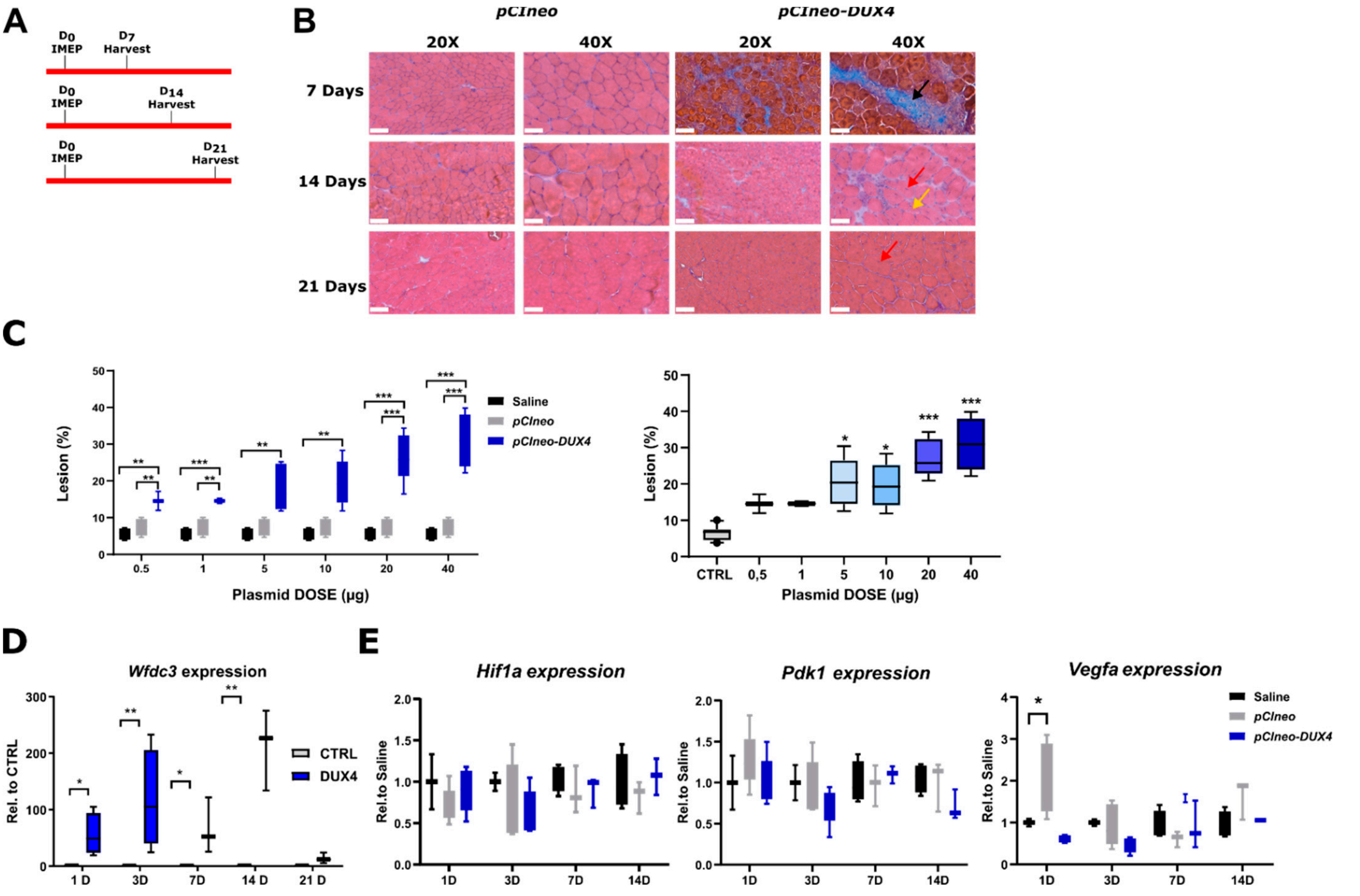

Figure S4. No effect of DUX4 expression on the Hif1α pathway in the DUX4 IMEP murine model with a low dose of *DUX4* expression. **(A)** Experiment time courses. **(B)** Representative sections of TA electroporated with 5 µg of *pCIneo* (left panel) or *pCIneo-DUX4* (right panel) plasmids at 7-, 14- and 21-days post-injection. ➔Fibrosis, ➔Central nuclei, ➔Atrophic fibers. Scale bar = 100µm for 20-X and 50 µm for 40-X magnification. **(C)** Dose–response of plasmid amount vs. muscle lesion area in mouse TA, 1-week post IMEP procedure. The lesion area percentage was evaluated on total cryosection of medial and proximal part of TA electroporated with different doses of a *DUX4* expression plasmid (*pCIneo-DUX4*). **Left panel:** ** $p < 0{,}01$, *** $p < 0{,}001$, Kruskal Wallis followed by Dunn's post hoc test. Saline: N=6, *pCIneo*: N=4, *pCIneo-DUX4*: 0,5 µg and 1µg: N=2, 5µg: N=6, 10 µg: N=7, 20µg: N=8, 40µg: N=4. **Right panel:** The results obtained from saline solution injected groups and *pCIneo* group were pooled into a single control group, as no statistical difference could be highlighted between groups at any time point. * $p < 0{,}05$, *** $p < 0{,}001$, Kruskal Wallis followed by Dunn's post hoc test. Control group: N=10, 0,5 µg and 1µg: N=2, 5µg: N=6, 10 µg: N=7, 20µg: N=8, 40µg: N=4. **(D)** Effect of *DUX4* expression on *Wfdc3* mRNA level in the IMEP model. RT-qPCR quantifications were normalized to *Rplp0*. * $p < 0{,}05$, ** $p < 0{,}01$; Kruskal Wallis followed by a Dunn's post-hoc test. For 1- and 3-day group: *pCIneo-DUX4*: N=4, *pCIneo*: N=6, saline: N=2. For 7- and 14-day groups: *pCIneo-DUX4*: N=3, *pCIneo*: N=3, saline: N=4. For the 21-day group: *pCIneo-DUX4*: N=3, *pCIneo*: N=4, saline: N=4. The results obtained from saline solution injected groups and *pCIneo* group were pooled into a single control group, as no statistical difference could be highlighted between groups at any time point. **(E)** Effect of DUX4 induction on *Hif1a*, *Pdk1* and *Vegfa* mRNA levels in the IMEP model. RT-qPCR normalized to *Rplp0* gene. * $p < 0{,}05$, Kruskal Wallis followed by a Dunn's post-hoc test. For 1- and 3-day group: *pCIneo-DUX4*: N=4, *pCIneo*: N=6. For 7- and 14-days groups: *pCIneo-DUX4*: N=3, *pCIneo*: N=4. For the 21-day group: *pCIneo-DUX4*: N=3, *pCIneo*: N=4. Results obtained from saline solution injected groups from all time points, were pooled together, as no statistical difference could be highlighted between groups at any time point for all tested genes, saline group: N=14.

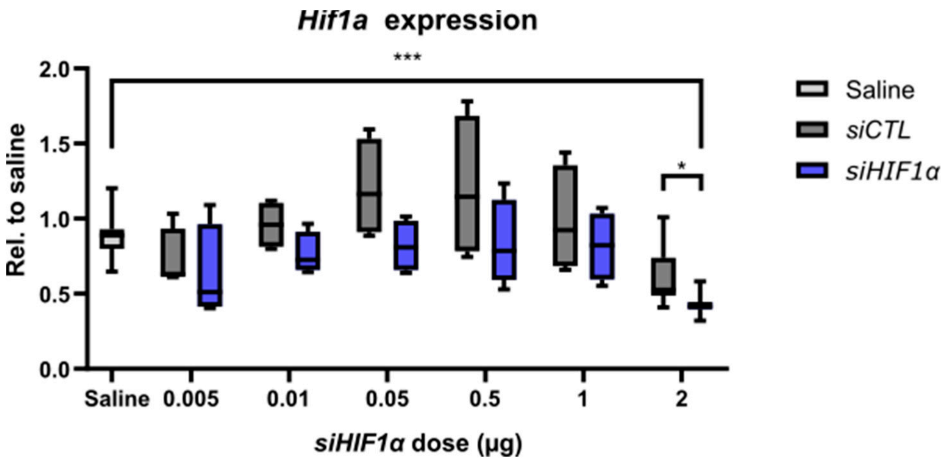

Figure S5. Efficiency of *siRNAs* directed against *Hif1a* mRNA (*siHIF1α*): dose-response analysis. The TA muscle was electroporated with either saline solution, *siCTL* or *siHIF1α*. *The Hif1a* mRNA level was quantified by RT-qPCR and normalized to *Rplp0*. * $p < 0.05$, *** $p < 0.001$, Kruskal Wallis followed by a Dunn's post-hoc test. Saline: N=10, 0.005 µg, 0.01 µg, 0.05 µg, 0.5 µg and 1 µg: N=4, 2µg : N=10.

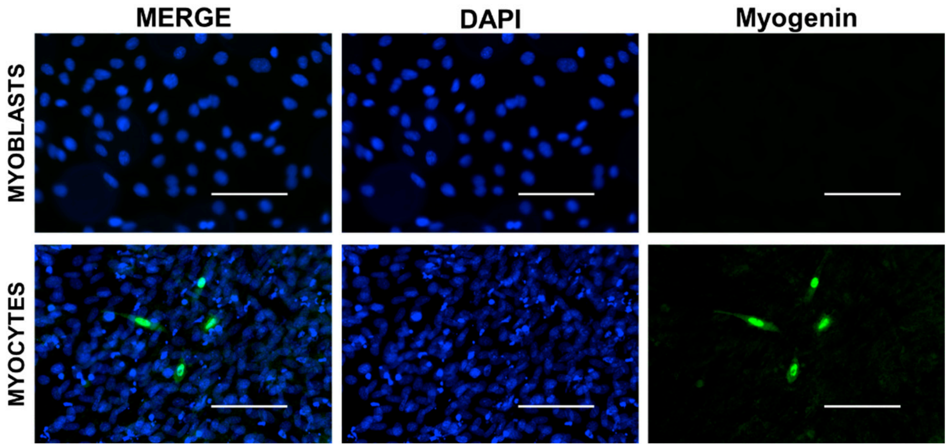

Figure S6. Mouse C2C12-iDUX4 myoblast differentiation into myocytes. **Myoblasts:** 25,000 cells were seeded per well in 24-well plates and grown at a standard PO_2 of 21%. 48h later, cells were fixed and Myogenin was detected by immunofluorescence (IF). **Myocytes:** 200,000 cells were seeded per well in 24-well plates coated with matrigel. 24h later, cells were switched to the differentiation medium for two days. Myogenin was detected by IF. Representative fields showing Myogenin$^+$ nuclei (green IF). Scale bar= 100µm
